# (*E*)-1-(2-Hy­droxy­phen­yl)-3-(2,4,5-trimeth­oxy­phen­yl)prop-2-en-1-one

**DOI:** 10.1107/S1600536811031382

**Published:** 2011-08-11

**Authors:** Hoong-Kun Fun, Thitipone Suwunwong, Kullapa Chanawanno, Pitikan Wisitsak, Suchada Chantrapromma

**Affiliations:** a X-ray Crystallography Unit, School of Physics, Universiti Sains Malaysia, 11800 USM, Penang, Malaysia; b Crystal Materials Research Unit, Department of Chemistry, Faculty of Science, Prince of Songkla University, Hat-Yai, Songkhla 90112, Thailand; c Excellence Center, Mae Fah Luang University, Thasud, Muang, Chaing Rai 57100, Thailand

## Abstract

In the title chalcone derivative, C_18_H_18_O_5_, the dihedral angle between the hy­droxy-substituted benzene ring and the trimeth­oxy-substituted benzene ring is 16.3 (1)°. The three meth­oxy groups are essentially coplanar with the benzene ring to which they are attached, with an r.m.s. deviation of 0.0208 Å. An intra­molecular O—H⋯O hydrogen bond generates an *S*(6) ring motif. In the crystal, weak C—H⋯O inter­actions link mol­ecules into helical chains along the *b* axis. These chains are connected into sheets parallel to the *bc* plane by further weak C—H⋯O inter­actions.

## Related literature

For background to and applications of chalcones, see: Boeck *et al.* (2006 ▶); Cheng *et al.* (2008 ▶); Hatayama *et al.* (2010 ▶); Jung *et al.* (2008 ▶); Lee *et al.* (2006 ▶); Liu *et al.* (2011 ▶); Nerya *et al.* (2004 ▶); Patil & Dharmaprakash (2008 ▶); Saydam *et al.* (2003 ▶); Tewtrakul *et al.* (2003 ▶). For related structures, see: Suwunwong *et al.* (2009 ▶); Fun *et al.* (2010 ▶). For the stability of the temperature controller used in the data collection, see: Cosier & Glazer (1986 ▶). For standard bond-length data, see: Allen *et al.* (1987 ▶). For hydrogen-bond motifs, see: Bernstein *et al.* (1995 ▶).
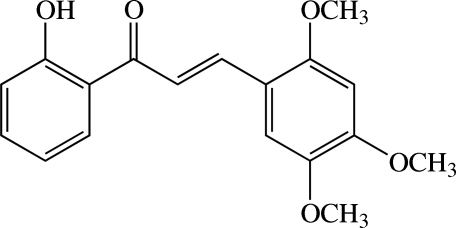


## Experimental

### 

#### Crystal data

C_18_H_18_O_5_
*M* *_r_* = 314.32
Orthorhombic, 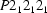

*a* = 4.2891 (2) Å
*b* = 17.3341 (9) Å
*c* = 20.5732 (10) Å
*V* = 1529.57 (13) Å^3^
*Z* = 4
Mo *K*α radiation
μ = 0.10 mm^−1^
*T* = 100 K
0.56 × 0.16 × 0.14 mm

#### Data collection

Bruker APEXII CCD area-detector diffractometer
Absorption correction: multi-scan (*SADABS*; Bruker, 2005 ▶) *T*_min_ = 0.946, *T*_max_ = 0.986
16077 measured reflections
2392 independent reflections
1946 reflections with *I* > 2σ(*I*)
*R*_int_ = 0.045

#### Refinement

*R*[*F* ^2^ > 2σ(*F* ^2^)] = 0.041
*wR*(*F* ^2^) = 0.095
*S* = 1.08
2392 reflections
280 parameters
All H-atom parameters refined
Δρ_max_ = 0.25 e Å^−3^
Δρ_min_ = −0.20 e Å^−3^

### 

Data collection: *APEX2* (Bruker, 2005 ▶); cell refinement: *SAINT* (Bruker, 2005 ▶); data reduction: *SAINT*; program(s) used to solve structure: *SHELXTL* (Sheldrick, 2008 ▶); program(s) used to refine structure: *SHELXTL*; molecular graphics: *SHELXTL*; software used to prepare material for publication: *SHELXTL* and *PLATON* (Spek, 2009 ▶).

## Supplementary Material

Crystal structure: contains datablock(s) global, I. DOI: 10.1107/S1600536811031382/lh5295sup1.cif

Structure factors: contains datablock(s) I. DOI: 10.1107/S1600536811031382/lh5295Isup2.hkl

Supplementary material file. DOI: 10.1107/S1600536811031382/lh5295Isup3.cml

Additional supplementary materials:  crystallographic information; 3D view; checkCIF report

## Figures and Tables

**Table 1:** Hydrogen-bond geometry (Å, °)

*D*—H⋯*A*	*D*—H	H⋯*A*	*D*⋯*A*	*D*—H⋯*A*
O1—H1*O*1⋯O2	0.89 (3)	1.73 (3)	2.541 (2)	152 (2)
C5—H5*A*⋯O5^i^	0.96 (2)	2.57 (2)	3.254 (3)	129.0 (18)
C16—H16*C*⋯O1^ii^	1.04 (3)	2.42 (3)	3.446 (3)	167.5 (18)

Symmetry codes: (i) 
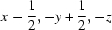
; (ii) 
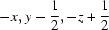
.

**Fig. 1.**
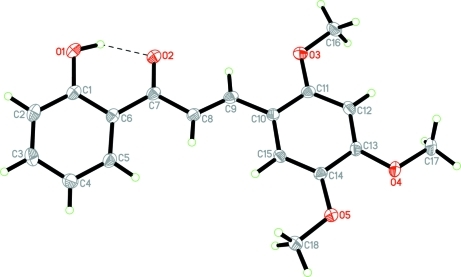
The molecular structure of the title compound, showing 50% probability displacement ellipsoids. An intramolecular O—H···O hydrogen bonds is shown as dashed line.

**Fig. 2.**
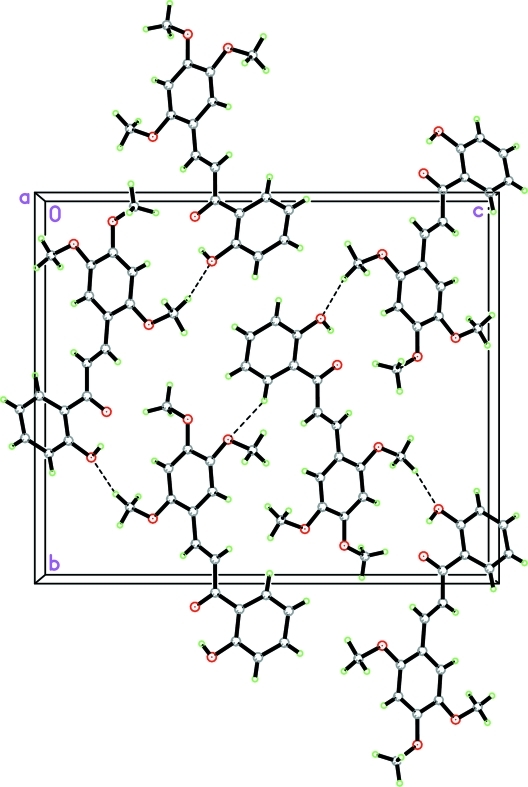
The crystal packing of the title compound, showing supramolecular sheets. Hydrogen bonds are shown as dashed lines.

## supplementary crystallographic information

### Comment

Chalcones or 1,3-diphenyl-2-propen-1-ones are commonly found in the natural products (Hatayama *et al.*, 2010). They have a wide range of applications including non-linear optical effects (Patil & Dharmaprakash, 2008) in fluorescent materials (Jung *et al.*, 2008) and have biological activities such as antibacterial (Liu *et al.*, 2011), anti-inflammatory (Lee *et al.*, 2006), antileishmanial (Boeck *et al.*, 2006), cytotoxic (Saydam *et al.*, 2003), anti-oxidant (Cheng *et al.*, 2008), HIV-1 protease inhibitory (Tewtrakul *et al.*, 2003) as well as anti-tyrosinase activities (Nerya *et al.*, 2004). The various interesting applications of chalcones lead us to synthesize the title chalcone derivative in order to study its tyrosinase inhibitory activity and also to compare its properies with the previously published related compounds (Suwunwong *et al.*, 2009; Fun *et al.*, 2010). Our experiment shows that (I) exhibits tyrosinase inhibitory activity with the IC_50_ value of 0.075 ± 0.000 mg ml^-1^. Its tyrosinase inhibitory activity is therefore about 0.08 times that of the standard anti-tyrosinase kojic acid. Herein the crystal structure of (I) is reported.

The molecule of (I) in Fig. 1 exists in an *E* configuration with respect to the C8═C9 double bond [1.343 (3) Å]. The molecule is twisted with the dihedral angle between the 2-hydroxyphenyl and the 2,4,5-trimethoxyphenyl benzene rings being 16.3 (1)°. The middle prop-2-en-1-one unit (O2/C7–C9) is slightly twisted with the torsion angle O2–C7–C8–C9 = -8.8 (3)°. The mean plane through this unit makes dihedral angles of 13.48 (14)° and 2.85 (14)° with the 2-hydroxyphenyl and the 2,4,5-trimethoxyphenyl benzene rings, respectively. The three methoxy groups of 2,4,5-trimethoxyphenyl unit are essentially co-planar with the attached benzene ring with torsion angles C16–O3–C11–C12 = -1.0 (3)°, C17–O4–C13–C12 = -2.5 (3)° and C18–O5–C14–C15 = 3.1 (3)°. An O1—H1O1···O2 intramolecular hydrogen bond generates an S(6) ring motif (Bernstein *et al.*, 1995) (Table 1). The bond distances have of normal values (Allen *et al.*, 1987) and are comparable with closely related structures (Suwunwong *et al.*, 2009; Fun *et al.*, 2010).

In the crystal packing (Fig. 2), weak C16—H16C···O1^ii^ interactions (Table 1) link the molecules into helical chains along the *b* axis. These chains are further connected by weak C5—H5A···O5^i^ interactions (Table 1) into supramolecular sheets parallel to the *bc* plane and stacked along the *a* axis.

### Experimental

The title compound was prepared by stirring the mixed solution of 2-hydroxyacetophenone (0.24 ml, 2 mmol) and 2,4,5-trimethoxybenzaldehyde (0.40 g, 2 mmol) in ethanol (30 ml) in the presence of 10% NaOH(aq) (5 ml). After 4 h of stirring at room temperature, the orange solid was obtained and was then collected by filtration, washed with distilled water, dried and purified by recrystallization from hot acetone. Yellow block-shaped single crystals suitable for *x*-ray structure determination were grown over a period of several days by slow evaporation of the acetone/ethanol (1:1 *v*/*v*) solvent at room temperature, Mp. 404–405 K.

### Refinement

All H atoms were located in difference Fourier maps and refined isotropically. The highest residual electron density peak is located at 0.78 Å from C14 and the deepest hole is located at 0.71 Å from C12. A total of 1662 Friedel pairs were merged before final refinement as there are no significant anomalous dispersion effects to determine the absolute configuration.

### Crystal data

**Table:**

C_18_H_18_O_5_	*D*_x_ = 1.365 Mg m^−^^3^
*M**_r_* = 314.32	Melting point = 404–405 K
Orthorhombic, *P*2_1_2_1_2_1_	Mo *K*α radiation, λ = 0.71073 Å
Hall symbol: P 2ac 2ab	Cell parameters from 2392 reflections
*a* = 4.2891 (2) Å	θ = 2.0–29.0°
*b* = 17.3341 (9) Å	µ = 0.10 mm^−^^1^
*c* = 20.5732 (10) Å	*T* = 100 K
*V* = 1529.57 (13) Å^3^	Block, yellow
*Z* = 4	0.56 × 0.16 × 0.14 mm
*F*(000) = 664	

### Data collection

**Table:**

Bruker APEXII CCD area-detector diffractometer	2392 independent reflections
Radiation source: sealed tube	1946 reflections with *I* > 2σ(*I*)
graphite	*R*_int_ = 0.045
φ and ω scans	θ_max_ = 29.0°, θ_min_ = 2.0°
Absorption correction: multi-scan (*SADABS*; Bruker, 2005)	*h* = −5→5
*T*_min_ = 0.946, *T*_max_ = 0.986	*k* = −23→17
16077 measured reflections	*l* = −24→27

### Refinement

**Table:**

Refinement on *F*^2^	Primary atom site location: structure-invariant direct methods
Least-squares matrix: full	Secondary atom site location: difference Fourier map
*R*[*F*^2^ > 2σ(*F*^2^)] = 0.041	Hydrogen site location: inferred from neighbouring sites
*wR*(*F*^2^) = 0.095	All H-atom parameters refined
*S* = 1.08	*w* = 1/[σ^2^(*F*_o_^2^) + (0.0466*P*)^2^ + 0.2277*P*] where *P* = (*F*_o_^2^ + 2*F*_c_^2^)/3
2392 reflections	(Δ/σ)_max_ = 0.001
280 parameters	Δρ_max_ = 0.25 e Å^−^^3^
0 restraints	Δρ_min_ = −0.20 e Å^−^^3^

### Special details

**Table:**

Experimental. The crystal was placed in the cold stream of an Oxford Cryosystems Cobra open-flow nitrogen cryostat (Cosier & Glazer, 1986) operating at 120.0 (1) K.
Geometry. All e.s.d.'s (except the e.s.d. in the dihedral angle between two l.s. planes) are estimated using the full covariance matrix. The cell e.s.d.'s are taken into account individually in the estimation of e.s.d.'s in distances, angles and torsion angles; correlations between e.s.d.'s in cell parameters are only used when they are defined by crystal symmetry. An approximate (isotropic) treatment of cell e.s.d.'s is used for estimating e.s.d.'s involving l.s. planes.
Refinement. Refinement of *F*^2^ against ALL reflections. The weighted *R*-factor *wR* and goodness of fit *S* are based on *F*^2^, conventional *R*-factors *R* are based on *F*, with *F* set to zero for negative *F*^2^. The threshold expression of *F*^2^ > σ(*F*^2^) is used only for calculating *R*-factors(gt) *etc*. and is not relevant to the choice of reflections for refinement. *R*-factors based on *F*^2^ are statistically about twice as large as those based on *F*, and *R*- factors based on ALL data will be even larger.

### Fractional atomic coordinates and isotropic or equivalent isotropic displacement parameters (Å^2^)

**Table:**

	*x*	*y*	*z*	*U*_iso_*/*U*_eq_	
O1	−0.2483 (4)	0.67956 (9)	0.11729 (7)	0.0294 (4)	
O2	0.0456 (4)	0.56059 (8)	0.15537 (6)	0.0277 (4)	
O3	0.5147 (4)	0.34185 (8)	0.25185 (6)	0.0239 (4)	
O4	0.3357 (4)	0.08107 (8)	0.17331 (7)	0.0241 (4)	
O5	−0.0186 (4)	0.13159 (7)	0.08358 (6)	0.0228 (4)	
C1	−0.3401 (5)	0.63667 (11)	0.06566 (9)	0.0205 (5)	
C2	−0.5266 (6)	0.67134 (12)	0.01880 (10)	0.0245 (5)	
C3	−0.6134 (6)	0.63167 (13)	−0.03616 (11)	0.0267 (5)	
C4	−0.5151 (6)	0.55587 (12)	−0.04557 (10)	0.0254 (5)	
C5	−0.3396 (5)	0.52027 (12)	0.00162 (10)	0.0214 (5)	
C6	−0.2498 (5)	0.55849 (11)	0.05878 (9)	0.0181 (4)	
C7	−0.0708 (5)	0.52102 (11)	0.11099 (9)	0.0194 (4)	
C8	−0.0393 (6)	0.43673 (11)	0.11163 (9)	0.0189 (4)	
C9	0.1472 (5)	0.40085 (12)	0.15445 (10)	0.0197 (5)	
C10	0.1985 (5)	0.31843 (11)	0.16063 (9)	0.0178 (4)	
C11	0.3882 (5)	0.28887 (11)	0.21024 (9)	0.0188 (4)	
C12	0.4385 (5)	0.20945 (12)	0.21658 (9)	0.0197 (5)	
C13	0.3018 (5)	0.15894 (11)	0.17306 (9)	0.0183 (4)	
C14	0.1069 (5)	0.18730 (11)	0.12307 (9)	0.0178 (4)	
C15	0.0587 (5)	0.26522 (11)	0.11777 (9)	0.0174 (4)	
C16	0.7030 (6)	0.31494 (14)	0.30485 (10)	0.0256 (5)	
C17	0.5405 (6)	0.04847 (13)	0.22131 (12)	0.0267 (5)	
C18	−0.2027 (6)	0.15760 (14)	0.03089 (11)	0.0236 (5)	
H2A	−0.586 (6)	0.7230 (14)	0.0249 (10)	0.033 (7)*	
H3A	−0.739 (7)	0.6549 (13)	−0.0687 (10)	0.029 (6)*	
H4A	−0.557 (6)	0.5303 (12)	−0.0823 (10)	0.021 (6)*	
H5A	−0.280 (6)	0.4677 (13)	−0.0057 (10)	0.029 (6)*	
H8A	−0.163 (6)	0.4083 (11)	0.0806 (9)	0.015 (5)*	
H9A	0.258 (6)	0.4335 (12)	0.1827 (9)	0.022 (6)*	
H12A	0.568 (6)	0.1911 (12)	0.2488 (9)	0.019 (6)*	
H15A	−0.079 (6)	0.2835 (12)	0.0829 (9)	0.020 (6)*	
H16A	0.766 (7)	0.3640 (14)	0.3280 (10)	0.036 (7)*	
H16B	0.890 (6)	0.2889 (13)	0.2889 (10)	0.028 (6)*	
H16C	0.574 (7)	0.2787 (14)	0.3348 (10)	0.033 (6)*	
H17A	0.467 (7)	0.0620 (13)	0.2662 (11)	0.040 (7)*	
H17B	0.757 (7)	0.0676 (13)	0.2147 (11)	0.031 (7)*	
H17C	0.539 (6)	−0.0083 (13)	0.2145 (9)	0.027 (6)*	
H18A	−0.274 (6)	0.1128 (13)	0.0074 (9)	0.023 (6)*	
H18B	−0.088 (6)	0.1914 (12)	0.0019 (10)	0.021 (6)*	
H18C	−0.394 (7)	0.1848 (14)	0.0468 (10)	0.032 (7)*	
H1O1	−0.123 (7)	0.6493 (15)	0.1401 (11)	0.042 (8)*	

### Atomic displacement parameters (Å^2^)

**Table:**

	*U*^11^	*U*^22^	*U*^33^	*U*^12^	*U*^13^	*U*^23^
O1	0.0418 (10)	0.0179 (8)	0.0286 (8)	0.0059 (8)	−0.0036 (9)	−0.0062 (7)
O2	0.0388 (10)	0.0186 (7)	0.0257 (7)	0.0033 (8)	−0.0077 (8)	−0.0047 (6)
O3	0.0308 (9)	0.0202 (7)	0.0207 (7)	−0.0013 (8)	−0.0069 (7)	−0.0022 (6)
O4	0.0311 (9)	0.0154 (7)	0.0256 (7)	0.0029 (7)	−0.0058 (7)	0.0032 (6)
O5	0.0307 (9)	0.0168 (7)	0.0208 (7)	0.0004 (7)	−0.0068 (7)	−0.0008 (5)
C1	0.0220 (11)	0.0172 (10)	0.0222 (10)	−0.0009 (9)	0.0066 (9)	0.0001 (8)
C2	0.0265 (12)	0.0155 (10)	0.0316 (11)	0.0015 (10)	0.0039 (10)	0.0023 (9)
C3	0.0269 (13)	0.0248 (12)	0.0283 (12)	0.0010 (10)	−0.0028 (11)	0.0085 (9)
C4	0.0311 (13)	0.0229 (11)	0.0221 (10)	−0.0027 (11)	−0.0029 (11)	0.0003 (9)
C5	0.0263 (12)	0.0151 (10)	0.0226 (10)	0.0002 (10)	0.0029 (10)	0.0003 (8)
C6	0.0188 (11)	0.0149 (10)	0.0206 (9)	−0.0012 (9)	0.0045 (9)	0.0019 (8)
C7	0.0211 (11)	0.0182 (10)	0.0189 (9)	−0.0001 (9)	0.0050 (9)	−0.0012 (8)
C8	0.0225 (11)	0.0167 (10)	0.0176 (9)	−0.0014 (9)	0.0023 (9)	−0.0011 (8)
C9	0.0223 (11)	0.0189 (10)	0.0178 (9)	−0.0022 (9)	0.0045 (9)	−0.0028 (8)
C10	0.0207 (10)	0.0181 (10)	0.0146 (9)	0.0022 (9)	0.0026 (9)	0.0001 (8)
C11	0.0204 (11)	0.0195 (10)	0.0164 (9)	−0.0009 (9)	0.0036 (9)	−0.0020 (8)
C12	0.0206 (11)	0.0228 (11)	0.0156 (9)	0.0023 (9)	−0.0003 (9)	0.0030 (8)
C13	0.0208 (11)	0.0153 (10)	0.0188 (9)	0.0013 (9)	0.0037 (9)	0.0034 (8)
C14	0.0205 (11)	0.0168 (10)	0.0162 (9)	−0.0006 (9)	0.0027 (9)	−0.0014 (8)
C15	0.0190 (10)	0.0193 (10)	0.0139 (9)	0.0015 (9)	0.0008 (9)	0.0021 (8)
C16	0.0287 (13)	0.0278 (13)	0.0202 (11)	−0.0017 (11)	−0.0052 (10)	0.0018 (9)
C17	0.0314 (14)	0.0196 (12)	0.0291 (12)	0.0059 (11)	−0.0038 (12)	0.0066 (9)
C18	0.0266 (13)	0.0217 (12)	0.0226 (11)	0.0003 (11)	−0.0066 (11)	−0.0003 (9)

### Geometric parameters (Å, °)

**Table:**

O1—C1	1.355 (2)	C8—C9	1.343 (3)
O1—H1O1	0.89 (3)	C8—H8A	0.96 (2)
O2—C7	1.246 (2)	C9—C10	1.451 (3)
O3—C11	1.368 (2)	C9—H9A	0.94 (2)
O3—C16	1.435 (3)	C10—C11	1.402 (3)
O4—C13	1.358 (2)	C10—C15	1.410 (3)
O4—C17	1.437 (3)	C11—C12	1.400 (3)
O5—C14	1.372 (2)	C12—C13	1.383 (3)
O5—C18	1.415 (3)	C12—H12A	0.92 (2)
C1—C2	1.389 (3)	C13—C14	1.413 (3)
C1—C6	1.417 (3)	C14—C15	1.371 (3)
C2—C3	1.375 (3)	C15—H15A	0.98 (2)
C2—H2A	0.94 (2)	C16—H16A	1.01 (2)
C3—C4	1.394 (3)	C16—H16B	0.98 (3)
C3—H3A	0.95 (2)	C16—H16C	1.04 (2)
C4—C5	1.375 (3)	C17—H17A	1.00 (2)
C4—H4A	0.89 (2)	C17—H17B	1.00 (3)
C5—C6	1.404 (3)	C17—H17C	0.99 (2)
C5—H5A	0.96 (2)	C18—H18A	0.96 (2)
C6—C7	1.471 (3)	C18—H18B	0.97 (2)
C7—C8	1.467 (3)	C18—H18C	1.00 (3)
			
C1—O1—H1O1	105.5 (16)	O3—C11—C12	122.76 (18)
C11—O3—C16	118.72 (16)	O3—C11—C10	116.12 (17)
C13—O4—C17	117.32 (16)	C12—C11—C10	121.11 (19)
C14—O5—C18	116.65 (16)	C13—C12—C11	119.82 (19)
O1—C1—C2	118.27 (18)	C13—C12—H12A	120.2 (13)
O1—C1—C6	121.59 (19)	C11—C12—H12A	119.9 (13)
C2—C1—C6	120.13 (19)	O4—C13—C12	125.57 (18)
C3—C2—C1	120.7 (2)	O4—C13—C14	114.32 (17)
C3—C2—H2A	120.9 (14)	C12—C13—C14	120.11 (17)
C1—C2—H2A	118.4 (14)	C15—C14—O5	125.95 (18)
C2—C3—C4	120.3 (2)	C15—C14—C13	119.32 (18)
C2—C3—H3A	121.4 (14)	O5—C14—C13	114.72 (17)
C4—C3—H3A	118.3 (14)	C14—C15—C10	122.06 (19)
C5—C4—C3	119.4 (2)	C14—C15—H15A	117.9 (12)
C5—C4—H4A	118.9 (15)	C10—C15—H15A	120.1 (12)
C3—C4—H4A	121.6 (14)	O3—C16—H16A	103.7 (14)
C4—C5—C6	122.0 (2)	O3—C16—H16B	111.0 (13)
C4—C5—H5A	117.5 (13)	H16A—C16—H16B	109 (2)
C6—C5—H5A	120.5 (13)	O3—C16—H16C	110.3 (14)
C5—C6—C1	117.40 (18)	H16A—C16—H16C	111.9 (18)
C5—C6—C7	123.12 (18)	H16B—C16—H16C	111 (2)
C1—C6—C7	119.48 (17)	O4—C17—H17A	110.4 (16)
O2—C7—C8	120.31 (18)	O4—C17—H17B	110.2 (14)
O2—C7—C6	120.05 (17)	H17A—C17—H17B	110 (2)
C8—C7—C6	119.61 (18)	O4—C17—H17C	106.8 (14)
C9—C8—C7	121.5 (2)	H17A—C17—H17C	111.1 (18)
C9—C8—H8A	121.7 (12)	H17B—C17—H17C	108 (2)
C7—C8—H8A	116.9 (12)	O5—C18—H18A	107.7 (13)
C8—C9—C10	127.1 (2)	O5—C18—H18B	112.3 (14)
C8—C9—H9A	115.3 (13)	H18A—C18—H18B	109.8 (17)
C10—C9—H9A	117.5 (13)	O5—C18—H18C	110.9 (13)
C11—C10—C15	117.57 (18)	H18A—C18—H18C	107 (2)
C11—C10—C9	120.77 (18)	H18B—C18—H18C	109.4 (19)
C15—C10—C9	121.66 (18)		
			
O1—C1—C2—C3	176.7 (2)	C16—O3—C11—C10	178.07 (19)
C6—C1—C2—C3	−3.1 (3)	C15—C10—C11—O3	−178.60 (18)
C1—C2—C3—C4	0.0 (3)	C9—C10—C11—O3	0.9 (3)
C2—C3—C4—C5	2.2 (3)	C15—C10—C11—C12	0.5 (3)
C3—C4—C5—C6	−1.4 (3)	C9—C10—C11—C12	179.9 (2)
C4—C5—C6—C1	−1.6 (3)	O3—C11—C12—C13	179.56 (19)
C4—C5—C6—C7	177.9 (2)	C10—C11—C12—C13	0.6 (3)
O1—C1—C6—C5	−176.0 (2)	C17—O4—C13—C12	−2.5 (3)
C2—C1—C6—C5	3.9 (3)	C17—O4—C13—C14	177.65 (18)
O1—C1—C6—C7	4.4 (3)	C11—C12—C13—O4	179.0 (2)
C2—C1—C6—C7	−175.7 (2)	C11—C12—C13—C14	−1.2 (3)
C5—C6—C7—O2	168.3 (2)	C18—O5—C14—C15	3.1 (3)
C1—C6—C7—O2	−12.2 (3)	C18—O5—C14—C13	−176.94 (19)
C5—C6—C7—C8	−13.8 (3)	O4—C13—C14—C15	−179.32 (19)
C1—C6—C7—C8	165.8 (2)	C12—C13—C14—C15	0.9 (3)
O2—C7—C8—C9	−8.8 (3)	O4—C13—C14—O5	0.7 (3)
C6—C7—C8—C9	173.2 (2)	C12—C13—C14—O5	−179.08 (19)
C7—C8—C9—C10	179.7 (2)	O5—C14—C15—C10	−179.87 (19)
C8—C9—C10—C11	−176.5 (2)	C13—C14—C15—C10	0.2 (3)
C8—C9—C10—C15	2.9 (3)	C11—C10—C15—C14	−0.8 (3)
C16—O3—C11—C12	−1.0 (3)	C9—C10—C15—C14	179.7 (2)

### Hydrogen-bond geometry (Å, °)

**Table:**

*D*—H···*A*	*D*—H	H···*A*	*D*···*A*	*D*—H···*A*
O1—H1O1···O2	0.89 (3)	1.73 (3)	2.541 (2)	152 (2)
C5—H5A···O5^i^	0.96 (2)	2.57 (2)	3.254 (3)	129.0 (18)
C16—H16C···O1^ii^	1.04 (3)	2.42 (3)	3.446 (3)	167.5 (18)

Symmetry codes:  (i) *x*−1/2, −*y*+1/2, −*z*; (ii) −*x*, *y*−1/2, −*z*+1/2.
